# Effects of Exercise and/or Diet Programs on Kinanthropometric and Metabolic Parameters in Obese Children: a Pilot Study

**DOI:** 10.2478/v10078-011-0041-x

**Published:** 2011-10-04

**Authors:** José M. Saavedra, Antonio Garcia-Hermoso, Yolanda Escalante

**Affiliations:** 1 Facultad de Ciencias del Deporte. AFIDES Research Group. Universidad de Extremadura, Spain.

**Keywords:** Body mass index, cholesterol, insulin, accelerometry

## Abstract

This study was aimed at determining the effects of implementing a medium-term (six-month) exercise and/or a diet program on the kinanthropometric and metabolic parameters of obese children. The participants were 42 subjects (27 boys, 15 girls), whose ages were between 8 and 11, divided into three groups according to the program they followed. The E group followed a physical exercise program (three 90-minute sessions per week), the D group a low calorie diet, and the E+D group both interventions. A repeated-measure ANOVA was used to compare measurements of the participants' kinanthropometric and metabolic parameters at different times of the program, with the means being compared using the Tukey post-hoc test. It was found that medium-term intervention based on the combination of exercise and low calorie diet improved the obese children's kinanthropometric and metabolic parameters, especially those related to the lipid profile. Also, this combined program was more effective in controlling weight than the exercise or low calorie diet interventions alone.

## Introduction

The prevalence of childhood obesity has been rising rapidly in recent years and is seen as a cause for alarm by public health agencies, health care clinicians, health care researchers and the general public (Barlow and Expert Committee, 2007). Using the standard *International Obesity Task Force* definition of pediatric obesity, the prevalence of obesity in children and young people aged 5–17 years worldwide is approximately 2–3% (Lobstein et al., 2004). Its impact on morbility, mortality and quality of life has made childhood obesity the epidemic of the XXI century (WHO, 2000) and a major public health problem (WHO, 2003). Childhood obesity is the commonest cause of such cardiovascular risk factors as hypertension, dyslipidemia and insulin resistance (Steinberger and Daniels, 2003), the principal components of metabolic syndrome in children and adolescents (Weiss et al., 2004). It is also an independent risk factor for obesity and increased health risks in adult life (Bibbins-Domingo et al., 2007).

Recommendations for caloric intake and physical activity need to be reassessed and better quantified at a population level because of the sedentary lifestyles of today's children (Han et al., 2010). For instance, recommendations regarding the treatment of childhood obesity focus typically on lifestyle changes, including increased physical activity and the promotion of healthy eating habits (Edmunds et al., 2001). An unbalanced diet and lack of physical activity cause excess fat storage in the child's adipose tissue (WHO, 2004) which in turn influences energy metabolism, insulin sensitivity (Kershaw and Flier, 2004), and other components of metabolic syndrome (Després et al., 2008). While weight loss through diet favourably modifies various cardiovascular risk indicators including the lipid profile (Nordmann et al., 2006), it appears that this treatment usually fails to raise high density lipoprotein cholesterol (HDL) levels in obese children (Woo et al., 2004). Since diet alone reduces both fat mass and muscle density, additional physical exercise seems necessary to counterbalance this latter decline (Sudi et al., 2001). Moreover, exercise improves insulin sensitivity (Kang et al., 2002) and blood lipid transport (Tolfrey et al., 2000), and is able to reverse the metabolic effect on the muscle of obese subjects (Tall, 2002). In this sense, there is evidence that physical activity and physical exercise programs during the years of growth may be protective against future cardiovascular disease (Rowland, 2001).

Despite extensive research on childhood obesity, there remains a lack of clear scientific evidence on the roles of diet and exercise in weight control, especially in children (Maffeis and Castellani, 2007). A meta-analysis (Leon and Sanchez, 2001) concludes that a diet combined with exercises will help reduce low density lipoprotein (LDL) and triglyceride (TG) levels, but it also decreases HDL cholesterol when compared with a purely exercise-based intervention. However, there is insufficient quality data to allow one treatment program to be recommended over another (Oude Luttikhuis et al., 2009). Other studies of obese children have shown that a combined diet and exercise intervention program lasting a relatively short time – two (Ben Ounis et al., 2008; Elloumi et al., 2009) or three (Shalitin et al., 2009) months – improved kinanthropometric (BMI and waist circumference) and metabolic (total cholesterol, LDL, and TG) variables. The aim of the present study was to determine the effects of implementing a medium-term (six-month) physical exercise and/or a diet program on the kinanthropometric and metabolic parameters of obese children.

## Methods

### Subjects

A total of 135 subjects were invited to participate through the collaboration of various schools in the town of Cáceres, Spain. The criterion for inclusion was that all participants had a body mass index (BMI) equal to or greater than the 97th percentile for the age and gender of the subject and ages between 8 and 11 years as defined by Spanish population curves (Hernández et al., 1988). Subjects were excluded if they: (i) regularly practised some physical activity or were following some other therapy (n=65); (ii) were involved in any weight control program (n=18); (iii) took any medication (n=8); or (iv) had any type of dysfunction limiting their physical activity (n=2). The final sample consisted of 42 subjects (10.2±1.1 years). They were divided into three groups: the E group who followed a multi-sports exercise program (n = 11, 10.7±0.9 years, 8 boys and 3 girls), the D group who followed a low calorie diet (n=16, 10.1±1.3 years, 10 boys and 6 girls), and the E+D group who followed a combination of the two programs – exercises and a low calorie diet (n=15, 10.1±1.0 years, 9 boys and 6 girls). There were no differences at the level of maturity whether between boys and girls of the same group (intragroup differences) or between boys and girls of the three groups (intergroup difference) (Table 1). This homogeneity at the stage of maturity allowed us to form groups with both sexes, as have previous studies (Reinehr et al., 2006; Chang et al., 2008). All the children's parents completed a prior informed consent form. The study was approved by the Bioethics Committee of the Universidad de Extremadura and respected the principles of the Declaration of Helsinki.

### Measurements

Kinanthropometric and metabolic parameters were evaluated for all subjects on the day before the intervention began, and at the beginning and end of the intervention. Pubertal stage was evaluated according to the Tanner classification (Tanner et al., 1966). The kinanthropometric measurements followed the ISAK protocol (Norton et al., 1996): body height, body weight, BMI, waist circumference, hip circumference, waist/hip ratio, and body fat mass (bio-impedance) (there were no values for the diet group, the instrumentation was unavailable during their evaluation). Standard equipment was used: a scale-mounted stadiometer (Seca, Berlin, Germany), weight scale (Seca, Berlin, Germany), bio-impedance analyzer (Bodistat, Isle of Man, Great Britain), and a non-extensible measuring tape (Holtain, Crymych, Great Britain). A blood sample (20 ml) was collected from an antecubital vein between 8:30 and 9:30 a.m. after an overnight fast. The metabolic parameters measured were: plasma total cholesterol concentration (Chod-Pad assay, automatic analyzer), HDL (HDL-C plus assay, automatic analyzer), triglyceridemia (Chod-Pad assay, automatic analyzer), blood glucose (glucose HK assay, automatic analyzer), insulinemia (human insulin RIA kit, Linco Research, Missouri, USA), and systolic (SBP) and diastolic (DBP) blood pressure (OMRON RX, Matsusaka, Japan). The homeostasis model assessment of insulin resistance (HOMA-IR) was used to measure the changes in insulin sensitivity among the subjects, and was calculated using the following formula (Matthews et al., 1985): fasting plasma insulin (μU/mL) × fasting plasma glucose (in mg/dL) ÷ 22.5 × 18.182. The following atherogenic indices were also calculated: LDL/HDL ratio and TC/HDL ratio.

### Intervention

Three interventions were tested: an exercise program (E group), a low calorie diet (D group), and the combination of the two (E+D group). The exercise program consisted of three weekly 90 min sessions. The sessions were held in a multi-sports hall, supervised by two MSc in Sports Science (AGH and ADM) and under the supervision of two PhD's (JMS and YE). The program comprised of a warm-up (15–20 min), a main part consisting of pre-sports and multi-sports games with a moderate to vigorous intensity aerobic component (60–65 min), and a cool-down (5–10 min). The intensity of the session was monitored by accelerometry to ensure that all the subjects performed the activities with the same intensity. A Caltrac accelerometer (Hemokinetics, Madison, WI, USA) was used, programmed to function as a physical activity monitor (Sallis et al., 1990). This uni-axial accelerometer contains a piezoelectric bender element which assesses the intensity of movement in the vertical plane. Its validity has been demonstrated as a method for estimating energy expenditure in children (Maliszewski et al., 1991), and has been used in other studies (Kimm et al., 2000; Moore et al., 2003; Sallis et al., 1990). A Caltrac accelerometer does not record such activities as rowing or swimming. However, no activity of this type was used either in the exercise program or in the subjects' daily physical activity for the duration of the study. The low-calorie diet consisted of five balanced meals spread throughout the day, with an energy intake of 1500 kcal/day. In this sense, there have been studies that recommend diets of between 1500 and 1800 kcal/d in obese children who are still growing (Braet et al., 2003; Flodmark, 2005) since in this way their growth and development are not compromised (Epstein et al., 1998). Thus the diet prescribed was of 1500 kcal/d, similar to that of other studies (Golan and Crow, 2004). This means a reduction of 400 kcal/d (22%), again a reduction similar to previous studies (Elloumi et al., 2009; Ben Ounis et al., 2008). The diet consisted of 57% carbohydrates, 17% proteins, and 26% fats. Foods were selected according to the subject’s dietary habits. A series of general recommendations were established focused on basic healthy lifestyle eating: consume ≥ 5 servings of fruits and vegetables every day; minimize sugar-sweetened beverages such as soft drinks, sports drinks, and sugar-added fruit juices; have more meals prepared at home rather than purchasing take-away restaurant food; etc.

### Statistical analysis

All the variables satisfied the tests of homoskedasticity (Levene homogeneity test) and normality (Kolmogorov-Smirnov test) of their distributions. The basic descriptive statistics (mean and standard deviation) were calculated. A repeated-measure ANOVA was used to compare the interaction between the different groups (E, D, E+D group) and different times of the test (preand post-test). The Tukey *post-hoc* test was used to compare means. Cohen's categories were used for the magnitudes of the effect size: small if 0≤|d|≤0.2; medium if 0.2<|d|≤0.5; and large if |d|>0.5 (Cohen, 1988). The level of significance for all statistical tests was set at p ≤ 0.05. All calculations were performed using SPSS (version 16.0).

## Results

There were no intergroup differences in the kinanthropometric and metabolic parameters before the program (Table 1).

There were differences in the kinanthropometric (Table 2) and metabolic (Table 3) parameters after the six-month interventions relative to baseline values in each group. While in the D group there were no significant changes in any of the kinanthropometric parameters, in the E group there were reductions in BMI (p=0.003) and body fat mass (p<0.001), and in the E+D group there were reductions in BMI (p<0.001), body fat mass (p=0.002), body weight (p=0.024), and waist (p<0.001) and hip (p<0.001) circumferences. With respect to the metabolic parameters, while the E group presented no change in any parameter, the D group presented reduced levels of TG (p=0.046), glucose (p=0.007), HOMA-IR (p<0.001), SBP (p<0.001), and DBP (p=0.050), and the E+D group presented improvements in HDL (p=0.038) and LDL (p=0.050) cholesterol, and in the LDL/HDL (p=0.009) and TC/HDL (p=0.004) ratios.

With respect to the group and time interaction in the kinanthropometric parameters (Table 4), differences were observed in body weight (D group>E+D group; p=0.041; ES=0.30) (E group>E+D group; p<0.001; ES=0.67) and hip circumference (E group>E+D group; p=0.004; ES=0.65). In the metabolic parameters (Table 5), there were differences in glucose (D group<E+D group; p=0.010; ES=−0.86), LDL/HDL ratio (E group>E+D group; p=0.007; ES=−0.03), and TC/HDL ratio (E group>E+D group; p=0.019; ES=−0.10).

## Discussion

**Figure 1:**
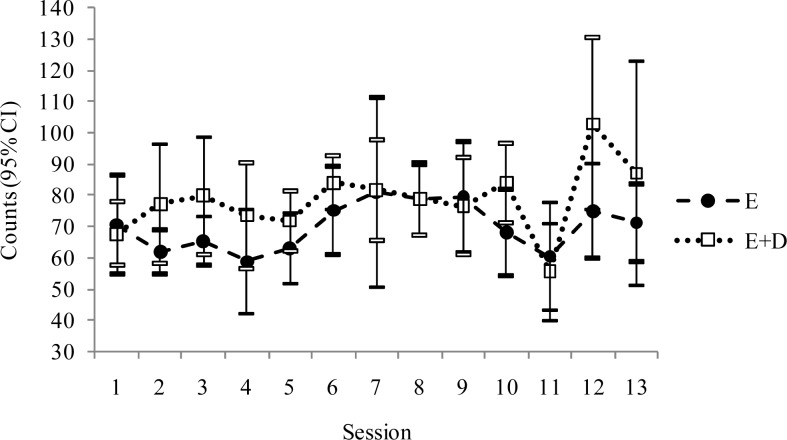
Representation of the mean and confidence intervals of the intensity of the sessions of the physical exercise program as evaluated by accelerometry. There were no differences between the E and E+D groups.

The present study has analyzed the medium-term (six months) effects on kinanthropometric and metabolic variables of an intervention based on exercise and/or a low calorie diet program. The mean participation of subjects in the exercise program was 81% (E group) and 83% (E+D group). Quantifying the intensities of 13 of the sessions selected at random showed no significant differences between the E and the E+D groups in any session, with a mean of 78.4 and 71.1 counts per session, respectively (Figure 1). Not all the sessions were quantified since the programming and placement of the accelerometers meant taking time away from the physical exercise program. The use of accelerometers allows one to objectively quantify the subjects' physical activity, ensuring that the intensity was similar in two groups. In developing treatment strategies for obesity, one requires quantitative information on physical activity to provide more effective goals (Kumahara et al., 2004), and, in so far as the methods used in the present work are concerned, this is the first study to have monitored physical activity sessions in an obese population using accelerometers.

### Intragroup differences

With respect to the intervention based solely on a physical exercise program (E group), there was a significant reduction in BMI (p=0.003) (Table 3). These findings, coherent with the literature (Chang et al., 2008; Shalitin et al., 2009), are indicative of the importance of physical exercise in regulating body weight. Not all the evidence points in the same direction, however, one published review suggests that exercise beneficially modifies body composition (fat and lean body mass) without changes in body weight or BMI in obese children (Watts et al., 2005). In this regard, there were improvements in body fat mass (E and E+D group, p<0.001 and p=0.002, respectively), confirming that aerobic exercise can positively influence body composition (Watts et al., 2005). Some studies, however, find no improvement in this parameter (Ben Ounis et al., 2008; Chang et al., 2008; Elloumi et al., 2009; Shalitin et al., 2009). There were no significant changes in any metabolic parameter for this group. The lack of improvement in TC and LDL levels was probably because the subjects maintained their normal intake of fats (Sudi et al., 2001). The results indicate that, once established, regular modest exercise can improve dyslipidemia (Chang et al., 2008). Although a short-term (three-month) intervention program (90 min/day for three days a week) has been reported as leading to significant improvements in TG concentrations (Shalitin et al., 2009), the initial values in that study were practically twice those of the present subjects. Neither did the E group's glucose levels improve, confirming that exercise alone is not always associated with changes in glucose metabolism (Watts et al., 2005).

The intervention based on a low calorie diet (D group) achieved no change in the kinanthropometric parameters after the six-month program (Table 2). Similar results were found in another study of shorter duration (six weeks), but of greater dietary restriction (between 900 and 1200 kcal/day) (Sung et al., 2002). In contrast, another intervention of longer duration (12 weeks) and similar diet (1200 kcal/day) significantly reduced the BMI and waist circumference (Shalitin et al., 2009). Regarding the metabolic parameters in the D group, there was a significant reduction in blood TG (p=0.046) and glucose (p=0.007). According to *The National Cholesterol Education Program*, a hypocaloric diet (30% or less of calories as total fat) reduces TC and LDL levels in normolipidæmic and hypercholesterolæmic subjects (Schaefer et al., 1995), with a smaller effect on TG and HDL (Turley et al., 1998). Presented results did not confirm these findings. Although there was a downward trend in these parameters after the intervention (Table 3), the subjects presented normal mean values (National Cholesterol Education Program, 1992). It is important to note that the TG concentration is influenced by, among other factors, the amount and quality of the carbohydrates and fats ingested, with the recommendation being to reduce the consumption of saturated fats and cholesterol, and of simple sugars or other carbohydrates of high glycæmic index (McKeown et al., 2004). Thus, the increased consumption of fruits and vegetables that were included in the diet could explain this reduction in TG (Law and Morris, 1999). The observed decrease in HOMA-IR (p<0.001), which is associated with improved insulin sensitivity (Reinehr et al., 2006) may also play a major role in reducing TG (Chang et al., 2008). Although such an improvement in insulin sensitivity has at times been associated with decreased body fat (Ribeiro et al., 2005), our data showed this improvement without any such significant decline. Finally, this intervention program (D group) reduced the blood pressure, both SBP (p<0.001) and DBP (p=0.050), suggesting that such blood pressure reduction is associated with sympathetic neurovascular attenuation (Ribeiro et al., 2005).

In the intervention which combined exercise and a low calorie diet (E+D group), improvements were observed in kinanthropometric parameters, with reductions in body weight (p=0.024), BMI (p<0.001), waist (p<0.001) and hip (p<0.001) circumferences, and body fat mass (p=0.002) (Table 2). These results are coherent with those of a previous study of less duration – two months (90 min/day for four days a week) and individual diet (−500 kcal/day below the initial dietary records) (Elloumi et al., 2009). Both studies show that a combined program of exercise and a low calorie diet contributes effectively to the prevention of obesity in children, probably because this strategy increases fat oxidation during exercise (Rodríguez and Moreno, 2006). Moreover, this intervention (E+D group) increased HDL (p=0.038), and decreased LDL (p=0.050) cholesterol and the LDL/HDL (p=0.009) and TC/HDL (p=0.004) ratios (Table 3), in agreement with the results of similar studies (Ben Ounis et al., 2008). In this regard, a combination therapy of diet plus exercise develops aerobic capacity, thus improving glucose tolerance and the lipoprotein profiles, and reducing the risk of coronary heart disease (Reinehr et al., 2006).

### Intergroup differences

The results showed that subjects benefited more from the combined program (E+D group) than from the physical exercise alone (E group) or the low calorie diet alone (D group) by better managing their body composition (Table 4). In particular, the E+D group achieved weight reduction relative to the D group (p=0.041; ES=0.30; 95% CI, −0.41 to 1.01), and even more markedly relative to the E group (p<0.001; ES=0.67; 95% CI, −0.14 to 1.47). This is a positive finding since a study (Watts et al., 2005) has suggested that a combination of dietary and exercise treatments individually designed for the specific pathological conditions of obese children facilitates the selective reduction of body fat, while maintaining the amount of muscle of the entire body. The E+D group also presented differences in hip circumference relative to the E group (p=0.004; ES=0.65; 95% CI, −0.15 to 1.45), showing that exercise alone does not consistently decrease body weight or fat distribution (Turley et al., 1998). With respect to metabolic parameters (Table 5), the glucose levels of the D group were lower than those of the E+D group (p=0.010; ES=−0.86; 95% CI, −1.60 to −0.12). No differences were observed, however, in insulin or HOMA-IR, although the values of both groups were within the normal range (Expert Committee on the Diagnosis and Classification of Diabetes Mellitus, 2003). The combined treatment (E+D group) led to greater changes than exercise alone (E group) in the LDL/HDL (p=0.007; ES=−0.03; 95% CI, −0.80 to 0.68) and TC/HDL (p=0.019; ES=−0.10; 95% CI, −0.88 to 0.68) ratios. It has been suggested that these indicators are better predictors of the reduction of cardiovascular disease risk than HDL, LDL, or TC values on their own (Natarajan et al., 2003), which could indicate that this combined treatment is more effective in controlling dyslipidemia than exercise alone. Although other studies do not in general observe these differences between one treatment and another (Oude Luttikhuis et al., 2009), the present results clearly show the importance of the dietary component in the treatment of subjects with childhood obesity (Shalitin et al., 2009).

### Limitations

A number of limitations of this study need to be kept in mind. First, there was a lack of initial randomization of the groups. Several subjects ate at the school's refectory, or were unable to attend the exercise program, making it impossible to randomly assign membership to one or another group. Nonetheless, the homogeneity of the groups was verified by the absence of initial differences in any of the variables (Table 1). Second, the duration of the intervention was only medium-term. Indeed, this was a pilot study for a subsequent longitudinal analysis of the parameters being monitored over the following three years, and then a follow-up study in the fourth year, an aspect of importance in children with this condition (Summerbell et al., 2005). Third, the sample was comprised of both boys and girls, although there were no intergroup or intragroup differences at the stage of maturity (Table 1) which allowed us to form groups with children of both sexes, since this choice would not affect the study. Finally, if the number of subjects had been greater the results would of course have been more convincing. However, the sample may be considered acceptable for the purposes of the present work since there have been studies in this area working with samples of similar sizes (Ribeiro et al., 2005; Ben Ounis et al., 2008; Elloumi et al., 2009).

## Conclusion

A medium-term intervention based on the combination of a low calorie diet and exercise in obese children showed improvements in kinanthropometric and metabolic parameters, especially in those related to the lipid profile. The interventions based on exercise alone or low calorie diet only achieved improvements in certain metabolic or kinanthropometric variables, respectively. The combined intervention, a low calorie diet plus exercise, was more effective in controlling body weight than either type of intervention alone. Similarly, there were no differences in effectiveness between the two interventions, exercise and low calorie diet, carried out alone, highlighting the need to combine the two to improve the kinanthropometric and metabolic parameters of obese children.

## Figures and Tables

**Table 1:** Pubertal status (mean ± SD) and intergroup (boys and girls) and intragroup (boys vs girls) differences

Pubertal status	E group (n=11)	D group (n=16)	E+D group (n=15)	Intergroup differences	
				F	p
Tanner stage – boys	1.88 ± 0.6	1.80 ± 0.6	2.00 ± 0.5	0.271	0.765
Tanner stage – girls	2.33 ± 0.6	1.83 ± 0.7	1.67 ± 0.8	0.790	0.476

Intragroup differences					
F-value, boys vs girls	1.604	0.135	0.371		
p-value, boys vs girls	0.237	0.719	0.553		

**Table 2:** Anthropometric characteristics of the study participants (mean and standard deviation pre- and post-test, inter- and intra-group differences)

Variable	E group (n=11)	D group (n=16)	E+D group (n=15)	Intergroup differences (p-value)
Height (m)				P
Baseline	1.49 ± 0.1	1.44 ± 0.1	1.47 ± 0.1	0.779
6 months	1.52 ± 0.1	1.47 ± 0.1	1.49 ± 0.1	0.283
Intragroup differences (p-value)	**<0.001**	**<0.001**	**<0.001**	
Weight (kg)				
Baseline	61.9 ± 10.2	58.9 ± 12.4	57.6 ± 11.2	0.501
6 months	62.7 ± 9.8	59.2 ± 13.6	55.3 ± 11.4	**0.008**
Intragroup differences (p-value)	0.168	0.745	**0.024**	
BMI, (kg/m^2^)				
Baseline	27.8 ± 3.5	28.5 ± 5.2	26.8 ± 3.5	0.106
6 months	27.0 ± 3.2	27.3 ± 5.6	24.7 ± 3.5	0.518
Intragroup differences (p-value)	**0.003**	0.300	**<0.001**	
Waist (cm)				
Baseline	94.7 ± 8.3	91.7 ± 10.9	90.7 ± 7.9	0.632
6 months	94.0 ± 7.8	89.8 ± 11.0	86.8 ± 9.2	0.120
Intragroup differences (p-value)	0.614	0.130	**<0.001**	
Hip (cm)				
Baseline	95.8 ± 8.3	97.4 ± 10.4	94.7 ± 8.1	0.488
6 months	96.6 ± 6.3	96.2 ± 10.2	91.2 ± 9.1	**0.006**
Intragroup differences (p-value)	0.459	0.176	**<0.001**	
Waist/hip				
Baseline	0.98 ± 0.15	0.94 ± 0.49	0.96 ± 0.35	0.181
6 months	0.97 ± 0.46	0.93 ± 0.42	0.95 ± 0.36	0.747
Intragroup differences (p-value)	0.205	0.264	0.175	
Body fat mass (%)				
Baseline	25.4 ± 6.92	-	25.7 ± 5.94	0.853
6 months	24.0 ± 6.41	-	22.4 ± 5.97	0.083
Intragroup differences (p-value)	**<0.001**	-	**0.002**	

The body fat mass data of the diet group were lost and hence could not be included in the analysis

**Table 3:** Metabolic characteristics of the study participants (mean and standard deviation pre- and post-test, inter- and intra-group differences)

Variable	E group (n=11)	D group (n=16)	E+D group (n=15)	Intergroup differences (p-value)
TC (mg/dl)				
Baseline	170.2 ± 15.2	173.6 ± 26.4	163.9 ± 21.9	0.432
6 months	169.8 ± 14.6	169.7 ± 20.9	158.0 ± 16.9	0.599
Intragroup differences (p-value)	0.920	0.215	0.153	
HDL (mg/dl)				
Baseline	51.0 ± 7.4	47.1 ± 12.0	41.8 ± 10.5	0.395
6 months	50.3 ± 11.3	48.4 ± 12.1	46.2 ± 14.5	0.148
Intragroup differences (p-value)	0.761	0.339	**0.038**	
LDL (mg/dl)				
Baseline	106.4 ± 16.7	108.1 ± 26.4	107.8 ± 14.6	0.315
6 months	107.2 ± 16.9	107.2 ± 20.1	98.1 ± 17.2	0.109
Intragroup differences (p-value)	0.811	0.654	**0.050**	
TG (mg/dl)				
Baseline	64.8 ± 27.5	85.6 ± 71.3	75.8 ± 50.5	0.569
6 months	61.5 ± 20.2	63.6 ± 28.9	64.5 ± 32.7	0.617
Intragroup differences (p-value)	0.920	**0.046**	0.394	
Insulin (μU/ml)				
Baseline	14.1 ± 8.3	21.9 ± 16.3	12.4 ± 7.9	0.308
6 months	11.4 ± 5.1	17.5 ± 13.7	9.1 ± 2.7	0.944
Intragroup differences (p-value)	0.202	0.320	0.123	
Glucose (mg/dl)				
Baseline	90.4 ± 5.5	86.8 ± 6.1	85.9 ± 4.9	0.422
6 months	90.4 ± 3.6	83.2 ± 5.5	88.1 ± 5.6	**0.034**
Intragroup differences (p-value)	1.000	**0.007**	0.302	
HOMA-IR				
Baseline	3.15 ± 2.1	3.49 ± 2.6	2.05 ± 1.8	0.331
6 months	2.53 ± 1.2	1.41 ± 2.6	2.42 ± 1.6	**0.011**
Intragroup differences (p-value)	0.208	**<0.001**	0.902	
LDL/HDL				
Baseline	2.12 ± 0.43	2.46 ± 0.99	2.69 ± 0.66	0.426
6 months	2.28 ± 0.82	2.39 ± 0.84	2.30 ± 0,70	**0.009**
Intragroup differences (p-value)	0.283	0.326	**0.009**	
TC/HDL				
Baseline	3.38 ± 0,46	3.94 ± 1.25	4.11 ± 0.87	0.479
6 months	3.54 ± 0.86	3.72 ± 1.02	3.63 ± 0.83	**0.025**
Intragroup differences (p-value)	0.319	0.141	**0.004**	
SBP (mm Hg)				
Baseline	111.8 ± 9.6	122.9 ± 8.9	117.9 ± 7.4	0.097
6 months	108.4 ± 11.2	111.4 ± 8.1	113.9 ± 7.4	0.157
Intragroup differences (p-value)	0.080	**<0.001**	0.197	
DBP (mm Hg)				
Baseline	65.3 ± 7.3	63.7 ± 7.9	66.9 ± 8.9	0.672
6 months	65.5 ± 8.2	57.0 ± 8.5	65.1 ± 6.1	0.410
Intragroup differences (p-value)	0.881	**0.050**	0.277	

TC, total cholesterol; HDL, high-density lipoprotein cholesterol; LDL, low-density lipoprotein cholesterol; TG, triglycerides; HOMA-IR, homœostasis model assessment index for insulin resistance; SBP=systolic blood pressure; DBP=diastolic blood pressure

**Table 4:** Differences between groups for the changes in anthropometric variables (mean and standard deviation, 95% confidence interval, and effect size)

Variable	E group (n=11)		D group (n=16)		E+D group (n=15)		Differences between groups					

							E vs D		E vs E+D		D vs E+D	

	Mean ± SD	95% CI	Mean ± SD	95% CI	Mean ± SD	95% CI	p	ES	p	ES	p	ES
Δ Weight (kg)												
Baseline - 6 months	0.9 ± 1.9	−0.4, 2.2	0.1 ± 3.1	−1.6, 1.7	−2.4 ± 2.5	−3.7, −1.0	0.69	0.3	**0.01**	**0.7**	**0.04**	**0.3**
Δ BMI (kg/m^2^)												
Baseline - 6 months	−0.6 ± 1.0	−1.3, −0.1	−1.3 ± 4.5	−3.7, 1.1	−2.1 ± 1.0	−2.7, −1.5	0.89	0.2	0.47	0.7	0.77	0.4
Δ Waist (cm)												
Baseline - 6 months	−0.6 ± 4.1	−3.4, 2.1	−1.9 ± 4.8	−4.4, 0.6	−3.9 ± 3.1	−5.6, −2.2	0.65	0.4	0.11	0.8	0.40	0.3
Δ Hip (cm)												
Baseline - 6 months	0.6 ± 3.7	−1.9, 3.1	−1.0 ± 3.5	−2.8, 0.8	−3.5 ± 2.6	−4.9, −2.0	0.22	−0.6	**0.01**	**0.7**	0.15	0.5
Δ Waist/hip (cm)												
Baseline - 6 months	−0.1 ± 0.1	−0.1, 0.1	−0.1 ± 0.3	−0.1, 0.1	−0.1 ± 0.2	−0.2, 0.0	0.81	0.1	0.75	0.1	0.99	−0.1
Δ Body fat (%)												
Baseline - 6 months	−1.4 ± 0.9	−2.0, −0.7	-	-	−3.3 ± 3.3	−5.2, −1.4	-	-	0.08	0.3	-	-

BMI, body mass index; CI, confidence interval; ES, effect size

**Table 5:** Differences between groups for the changes in metabolic variables (mean and standard deviation, 95% confidence interval and effect size)

Variable	E group (n=11)		D group (n=16)		E+D group (n=15)		Differences between groups					

							E vs D		E vs E+D		D vs E+D	

	Mean ± SD	95% CI	Mean ± SD	95% CI	Mean ± SD	95% CI	p	ES	p	ES	P	ES
Δ TC (mg/dl)												
Baseline - 6 months	0.0 ±12.1	−8.1, 8.1	−4.6 ± 16.4	−13.3, 4.2	−5.9 ± 11.6	−12.3, 0.6	0.79	0.1	0.50	0.7	0.84	0.6
Δ HDL (mg/dl)												
Baseline - 6 months	−1.1 ± 6.7	−5.6, 3.4	1.6 ± 5.8	−1.5, 4.7	4.4 ± 6.5	0.6, 8.1	0.73	0.2	0.22	0.3	0.53	0.2
Δ LDL (mg/dl)												
Baseline - 6 months	0.8 ± 11.1	−6.6, 8.3	−0.8 ± 14.6	−8.6, 9.0	−9.8 ± 11.72	−17.2, 2.3	0.94	0.01	0.13	−0.1	0.18	−0.1
Δ TG (mg/dl)												
Baseline - 6 months	−3.3 ± 25.4	−20.4, 13.8	−22.0 ± 56.0	−51.9, 7.9	−11.3 ± 52.6	−43.1, 20.5	0.59	−0.1	0.91	−0.5	0.83	−0.4
Δ Insulin (μU/mL												
Baseline - 6 months	−2.6 ± 6.4	−6.9, 1.7	−4.5 ± 8.3	−13.1, 4.2	−3.3 ± 6.9	−8.3, 1.6	0.87	−0.5	0.97	−0.3	0.95	0.5
Δ Glucose (mmol/L)												
Baseline - 6 months	0.0 ± 4.6	−3.1, 3.1	−3.6 ± 5.2	−6.4, −0.9	2.2 ± 5.0	−0.9, 5.2	0.17	1.5	0.55	0.1	**0.01**	**−0.9**
Δ HOMA-IR												
Baseline - 6 months	−0.8 ± 1.4	−1.7, 0.2	−2.0 ± 1.7	−2.9, −1.0	0.1 ± 2.5	−1.3, 1.6	0.84	0.5	0.99	0.3	0.78	−0.3
Δ LDL/HDL												
Baseline - 6 months	0.2 ± 0.5	−0.1, 0.6	−0.1 ± 0.4	−0.3, 0.1	−0.4 ± 0.3	−0.8, −0.2	0.35	−0.1	**0.01**	**−0.1**	0.12	−0.4
Δ TC/HDL												
Baseline - 6 months	0.2 ± 0.5	−0.1, 0.6	−0.3 ± 0.7	−0.6, 0.1	−0.48 ± 0.39	−0.7, −0.3	0.21	−0.2	**0.02**	**−0.1**	0.41	−0.3
Δ SBP (mm Hg)												
Baseline - 6 months	−3.4 ± 5.7	−7.2, 0.5	−11.4 ± 10.2	−16.9, −6.0	−3.9 ± 10.6	−9.8, 1.9	0.09	−0.3	0.99	−1.0	0.08	−0.8
Δ DBP (mm Hg)												
Baseline - 6 months	0.3 ± 5.9	−3.7, 4.2	−6.7 ± 11.1	−12.6, −0.8	−1.74 ± 6.6	−5.4, 1.9	0.11	1.0	0.82	−0.2	0.23	−1.3

TC, total cholesterol; HDL, high-density lipoprotein cholesterol; LDL, low-density lipoprotein cholesterol; TG, triglycerides; HOMA-IR, homœostasis model assessment index for insulin resistance; SBP, systolic blood pressure; DBP, diastolic blood pressure; CI, confidence interval; ES=effect size
